# The Relationship between Inflammatory Factors, Hemoglobin, and VO2 Max in Male Amateur Long-Distance Cross-Country Skiers in the Preparation Period

**DOI:** 10.3390/jcm13206122

**Published:** 2024-10-14

**Authors:** Natalia Grzebisz-Zatońska

**Affiliations:** Department of Human Biology, Faculty of Physical Education, Józef Piłsudski University of Physical Education in Warsaw, 00-968 Warsaw, Poland; n.grzebisz@gmail.com

**Keywords:** VO2 max, hematological determinants, long-distance cross-country skiing, amateur, general preparation period, C-reactive protein

## Abstract

**Background**: Identifying factors affecting heart health in amateur athletes can significantly impact their health and help them achieve high performance. The current knowledge of these predictors is insufficient. The purpose of this study was to identify the biochemical determinants of maximal oxygen uptake (VO2 max) in male amateur long-distance cross-country skiers (37.9 ± 6.58 years, 51.08 ± 4.61 VO2 max ml/kg/min) in the preparation period. **Methods**: In this cross-sectional study, a time trial test was used to determine VO2 max and venous blood via biochemical markers. Descriptive statistics and Pearson correlation were used to analyze the data. The regression model determined the predictors. **Results**: VO2 max was significantly correlated with nine moderate or weak variables. Two regression models (*R*^2^ = 0.94 and *R*^2^ = 0.9) each identified two determinants of VO2 max, hemoglobin (*p* < 0.001) and C-reactive protein (*p* < 0.001), as well as erythrocyte sedimentation (*p* < 0.001) and platelets (*p* = 0.03). Only hemoglobin positive affected VO2 max. **Conclusions**: The results may indicate, in addition to results regarding hemoglobin concentration and its changes, the necessity to monitor the immune system, which may affect the capacity for amateur exercise. Biochemical monitoring is an essential tool for evaluating the individual adaptation to exercise and developing an effective training plan. The application of this knowledge can facilitate the achievement of optimal individual performance capabilities among cross-country skiing amateurs.

## 1. Introduction

The role of physical training is to adapt to exercise, which allows for the maximum use of physiological reserves. For cross-country skiers (xc skiers), the most important part of the annual training cycle is the preparation period and its basic sub-period in the summer months. Professional athletes, as well as amateurs who later compete in marathons and ultramarathons, perform mostly low- and moderate-intensity endurance, specialized endurance, and strength training, especially bodybuilding circuit training. Subsequently, the aforementioned efforts are carried out, particularly in the medium-intensity zone, wherein the utilization of metabolites produced in the muscles is feasible (they are buffered and the acid–base balance is maintained). Training in this zone can be continued for a longer period. Training at such an intensity enhances overall endurance. Loads are also carried out in the high-intensity zone, during which the body is still able to maintain acid-base balance, although for a shorter time (30–45 min). These are efforts of a repetitive or variable nature, and their energy comes from both the aerobic and anaerobic metabolism. Training in this zone significantly enhances overall endurance. The control and pre-start periods that follow are characterized by reduced effort volume and higher intensity [1]. Excessive workload and a lack of recovery in the preparation period, together with an increased number of illnesses in the population during the autumn period, can contribute to lower immunity in the later period, which affects sports performance and can disrupt the sports preparation cycle. In endurance sports, the indicator used to assess exercise potential is the maximum oxygen uptake (VO2 max), which increases in response to appropriate training stimulation [2,3]. Many factors determine VO2 max, for example, respiratory functions, blood circulation, metabolism, and muscle flow. Another group relates to hematological and biochemical indices. An appropriate change in their values can indicate adaptation to exercise and protect against overuse injuries, overtraining, and their consequences. Although injuries are rare among xc skiers, overuse injuries and poor training adaptations can affect health and training results [4]. The effect of this type of exercise on heart function is also noted. Swedish male frequent participants of the prestigious Vasaloppet race (90 km) were more prone than nonskiers to bradycardia and implantations of a pacemaker [5].

Monitoring physiological, morphological, and anthropometric variables or mental state can help prepare an appropriate training plan to support the progression of exercise capacity. This is particularly important in the group of middle-aged amateurs, who are non-professionals, and active professionals, who usually have family responsibilities and limited time for training, and compete in marathons and ultramarathons. They are usually less often supported by physiotherapists and doctors than professional athletes. This influences the lack of adequate recovery after training and starting, and potentially causes negative changes in the body in response to the effort. The risk of heart attack is higher among middle-aged amateur skiers because of the lack of collateral circulation. Demanding exercise such as a ski marathon and inappropriate training can contribute to this risk. Well-planned training can mitigate the risk and improve fitness and adaptation to the effort.

The determinants of VO2 max in elite skiers are thoroughly researched [6,7,8,9,10].

It was indicated that the weakening of the immune system was a common phenomenon, which significantly reduced exercise capacity [7]. This can also be easily observed in practice. The parameters of the red blood cell system, including hemoglobin, significantly affect VO2 max. An increase in hemoglobin is associated with better oxygen transport from blood to cells and is an important factor in determining exercise capacity in long-term efforts [6]. In the group of professional athletes, biomechanical determinants were also assessed. The assessment of technique and economy was conducted, yet no correlation was established between the findings and biochemical parameters.

However, knowledge of these changes in middle-aged amateurs is still limited. Few results describe the relationship between exercise, health, and exercise capacity in amateurs [3,11,12]. The frequency and duration of coughing and the possibility of asthma in cross-country skiers were assessed [12]. The impact of the immune system on exercise capacity was discussed by this researcher. However, negative changes were evaluated using indirect methods (a questionnaire). Furthermore, the assessment of VO2 max was conducted across various age groups of cross-country skiers so as to identify the impact of training on performance parameters [3,11]. Nevertheless, there is still a lack of data on biochemical markers, their changes, and their influence on VO2 max in the group of cross-country skiing amateurs. The growing popularity of marathon and ultramarathon efforts, along with a need to assess biochemical and physiological parameters and predictors of demonstrated exercise capabilities, makes it essential to collect data. Currently, there is a lack of such information.

This study aimed to identify the biochemical determinants of maximal oxygen uptake (VO2 max) in male amateur long-distance cross-country skiers in the preparation period. Participants in marathons and ultra-marathons are particularly exposed to overloads related to physical effort. The systematic monitoring of biochemical and physiological parameters can be used to assess the body’s adaptation to effort. This is essential in the training process, including for amateurs. Furthermore, the analysis of variables can indicate factors that significantly affect the VO2 max value. The assessment of the scope of these changes can therefore be beneficial in achieving higher exercise capacities for amateurs.

## 2. Materials and Methods

### 2.1. Participants

Sixteen male amateur cross-country skiers participated in the study. They lived in a large city and were employed. The study was approved by the Bioethics Committee of the Faculty of Human Nutrition and Consumption at the Warsaw University of Life Sciences (SGGW) (No. 38p/2018, approved on 22 January 2019) and conducted via the Good Clinical Practice guidelines and the Declaration of Helsinki. Informed consent was obtained from all subjects involved in the study.

### 2.2. Selection Criteria

Inclusion criteria were the completion of at least three long-distance ski races during the last 12 months, physical training of up to 90 min per day, medical clearance, and signing the document with permission to participate in the study. Lack of written and medical consent and any illness were exclusion criteria.

Participation in the study was voluntary. Information about it was disseminated via social media and the organizers of the main cross-country skiing events in the country of study. Information about the study was also shared by the volunteers themselves with their amateur colleagues.

It was essential to meet the inclusion and exclusion criteria. Therefore, after giving their willingness to participate, these were checked. If they were met, the person was included in the study.

The amateurs respected the rules of the World Anti-Doping Agency (WADA), which is also a key requirement during sports competitions, including Worldloppet races, organized under the auspices of the International Ski and Snowboard Federation (FIS).

### 2.3. Study Design

The purpose of the time trial test was to assess VO2 max (mL/kg/min). The cross-sectional study was performed in September, after the general preparation and before the control and pre-start periods. Using the HP Cosmos CPET treadmill (Nussdorf-Traunstein, Germany) and the Cosmed Quark/k4B2 portable gas analysis system (Rome, Italy) the speed was increased every 180 s by 1 km/h and the incline by 1% from the initial speed of 6 km/h and no incline. The test was executed until the subjective feeling of exhaustion. Determination of VO2 max was associated with a cessation of the increase in oxygen uptake (plateau) or a significant slowdown in its increase despite the increasing exercise load. Heart rate was recorded with a Garmin ANT+ heart rate monitor (Olathe, KS, USA). The test was conducted in laboratory conditions with a temperature range of 19–21 °C and a relative humidity range of 40–50%. The maximal results of the test are presented in this article.

### 2.4. Anthropometric Measurements

The Tanita Body Composition Analyzer BODY IN MC-980 MA (Tokyo, Japan) was used to measure body composition and weight right before the time trial. The analyzer consists of an eight-point touch electrode system. Measurements included body weight, fat mass (% and kg), and BMI (body mass index).

### 2.5. Biochemical Parameters

Venous blood samples were obtained between 7 and 10 AM on an empty stomach and before the time trial test. Sodium, potassium, C-reactive protein, and lipid profile (total cholesterol, high-density lipoprotein cholesterol (HDL-C), low-density lipoprotein cholesterol (LDL-C), and triglycerides) were measured with the spectrophotometric method, and cortisol, testosterone, and thyroid stimulating hormone (TSH) with the electrochemiluminescence immunoassay (ECLIA). ESR (erythrocyte sedimentation rate) was measured using Alifax (Polverara, Italy) and the automated method. Cobas 8000 (Basel, Switzerland) and spectrophotometric methods measured magnesium, creatinine, urea, iron, uric acid, total calcium, alanine aminotransferase (ALT), amylase, aspartate aminotransferase (AST), gamma-glutamyl transpeptidase (GGTP), alkaline phosphatase (ALP), glucose and total bilirubin.

This article presents only data that showed correlations with VO2 max and were significant for regression. All results can be made available after consultation with the author.

### 2.6. Statistics

The described statistics were the number of persons (N), standard deviation (SD), and arithmetic mean, minimum (Min), maximum (Max), and median. The Shapiro–Wilk test and Pearson correlation were then applied. The statistical significance of the results can be interpreted as shown in Table 1.

A methodology was employed whereby potential models of the relationship between VO2 max and variables that are correlated with it were constructed. Each model was constructed with two dependent variables. The quality of the models was evaluated using the coefficient of determination (*R*^2^).

This indicated the degree of fit of the regression function to the empirical data. Based on the analysis of the correlation coefficients, the independent variables were selected for inclusion in the model, and their potential combinations were verified. The combinations with the highest *R*^2^ were presented as the optimal models.

Consequently, the *R*^2^ value determined the proportion of the variance of the explained variable that was explained by the regression function, within the range of 0–1 (0–100%). The explanatory power of the model was reflected in the magnitude of the *R*^2^ value. A fit index of 1 indicates a model that is perfectly fitted.

The prevalence of models exhibiting a markedly elevated *R*^2^ coefficient in this study suggested the potential for overfitting. Accordingly, the data were partitioned into a training set (comprising 70% of the data) and a test set (comprising 30% of the data). This is a solution that is employed when it is not feasible to generalize data sets. This enabled us to evaluate the model’s performance on each data set, identify potential overfitting, and assess the statistical process of training. The degree of accuracy observed in both data sets was used as a measure of performance, to infer the presence of overfitting.

The coefficients thus obtained were employed in the prediction of VO2 max values for variables drawn from the test set. A comparative analysis was performed on the predicted values and observed values from the test set, employing the Mean Square Error (MSE). Two models were identified as optimal for minimizing MSE for the test set and the entire data set, respectively.

The models constructed previously were thus selected based on their *R*^2^ coefficients, which exceeded 0.9. The models were rebuilt for the variables used, this time based on data from the training set only. This was because the model performed better on the training set than on the test set, indicating probable overfitting.

Statistically significant differences were noted at *p* ≤ 0.05.

The calculations were performed in statistical software (ver. 3.6.0) (Chicago, IL, USA).

## 3. Results

### 3.1. The Somatic Variables

Table 2 shows the somatic variables and maximal oxygen uptake.

### 3.2. Correlations for Independent Variables

Nine variables were significantly correlated with VO2 max—most of them moderate or weak and negative. The values of each were within established norms for the population. Table 3 presents only statistically significant results.

### 3.3. Regression Model

Models of the relationship between variables correlated with VO2 max were constructed, with two dependent variables in each model. The data are presented in Table 4 and Table 5.

**Figure 1:**
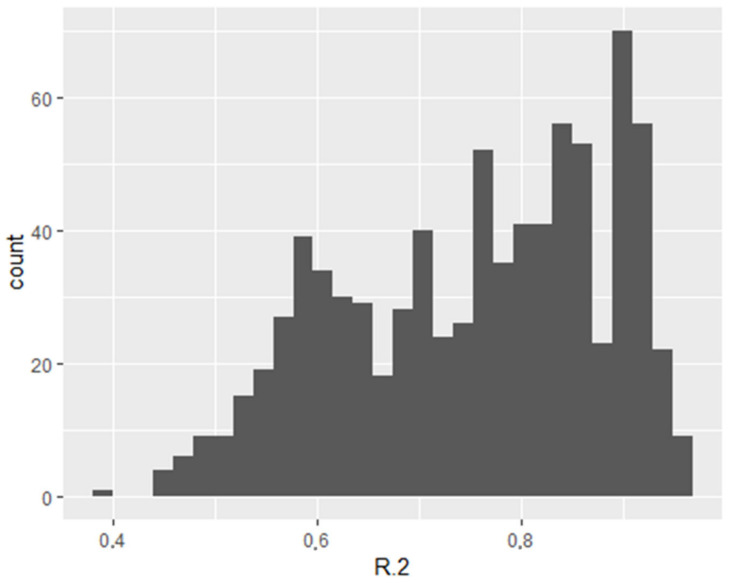
*R*^2^ value histogram for models for VO2 max. The Y-axis shows the number of models where the given *R*^2^ value was obtained.

The quality of each model was evaluated using *R*^2^, as shown in the *R*^2^ histogram below (Figure 1).

Statistically significant differences were noted at *p* ≤ 0.05.

## 4. Discussion

Maximum oxygen uptake is an important factor in determining exercise capacity. It is often assessed in a group of professional athletes. In the case of amateurs competing in marathons and ultramarathons, such results are still lacking. Gender, age, genetic predisposition, and biochemical variables are among the factors that influence its value. This study aimed to identify the biochemical determinants of maximal oxygen uptake (VO2 max) in amateur long-distance cross-country skiers during the preparation period.

### 4.1. Correlations

The correlation coefficient identifies whether a change in one indicator is observed when the value of another increases or decreases. The study did not reveal any strong correlations, indicating that deviations are to be expected. The findings may, however, be employed to evaluate the health status of amateurs and may also provide insights for management.

The study observed a positive, moderate correlation between leukocytes, hemoglobin, and hematocrit. A very weak, positive correlation was observed in erythrocytes.

It is well documented that the above variables of the erythrocyte system have a beneficial influence on VO2 max [13,14,15].

A reduction in hemoglobin concentration may be the result of excessive training, inflammation, an inadequate diet, or the shedding of red blood cells, for example during running. A minor decline in hematocrit resulting from training is also attributable to an increase in plasma volume. This does not indicate clinical anemia, but is a transient phenomenon observed in athletes [16,17,18].

The study by Bent R. Rønnestad et al. [19] evaluated the impacts of five thermal suit training sessions per week on hemoglobin mass—it increased after five weeks (the same thing applied to red blood cells) compared to the control group. Nevertheless, no changes were observed in the endurance performance.

The addition of altitude training influences the formation of blood cells and physiological adaptations to prolonged exercise. This has been demonstrated to enhance the VO2 max of cross-country skiers and cyclists [20,21,22,23].

An increase in leukocyte count is a clear indicator of improved immune function. Conversely, periods of intense, prolonged training have been shown to reduce the outcome [24]. Factors that weaken immunity and increase susceptibility to infection include insufficient sleep, inadequate recovery time, mental stress, and inadequate nutrition, particularly deficiencies in protein and essential micronutrients. Although leukocyte count is not a predictor of VO2 max in these studies, it showed correlations, indicating that higher levels of training may result in a stronger immune response. The U-shaped curve also explains this phenomenon. It indicates that both excessive training loads and inactivity have a detrimental effect on the body’s immune system [25].

Studies have indicated that maintaining high levels of aerobic fitness throughout the natural aging process can help prevent the buildup of aging T cells and support immune function [26]. Aging negatively affects natural immunity and increases the risk of infections in old age. Studies show that physical activity can mitigate this risk without side effects. This is an additional benefit that can result from amateur activity [27].

A significant increase in leukocytes occurs after a single, intense exercise, during intense stress, and in viral, fungal, and bacterial infections. Regular, moderate exercise reduces the frequency of infection, but prolonged, intensive workouts cause a transient reduction in various aspects of immune function. Michael Gleeson indicated [28] that this may involve—among others—neutrophil respiratory burst and the proliferation of lymphocytes that can last up to 24 h post-exercise.

While there is a lack of studies showing the correlation between leukocytes and VO2 max, their indirect effect on this parameter can be explained. The better the body functions, the lower the number of aging lymphocytes, and the stronger the immune response. This then leads to a reduced risk of infections, fewer interruptions to training, and better support for increased performance [29,30,31].

The decrease in VO2 max in these studies can be equated with a decrease in ESR (mm/h) and Gamma-glutamyl transferase (GGTP) (U/L), although the correlation was moderate. A weak but statistically significant correlation was shown for platelets (thou/µL), procalcitonin (PCT) %, and C-reactive protein (CRP) (mg/dL). An elevated erythrocyte sedimentation rate (ESR) (mm/h) is an indicator of chronic inflammation in the body and poor recovery. Higher gamma-glutamyl transferase activity is a clear sign of disease and inflammation. Furthermore, the inflammatory process results in the production of platelets and higher amounts of CRP and activated PCT. The results of this study are consistent with reports from other researchers [32,33].

This is the correct and desired response to inflammation. It leads to recovery and a return to homeostasis. It is crucial to prepare athletes for this state and ensure super-compensation during the preparation period. However, over an extended period, it will inevitably lead to a reduction in exercise capacity, as measured by VO2 max, when illness, exhaustion, or overtraining occurs. This is in line with the findings of other researchers [34,35,36,37,38].

It is therefore crucial to implement a comprehensive monitoring program to track alterations in the immune system and evaluate the influence of physical exercise on these changes. The reduction post-exercise of factors that enhance temporary immune depression (proper nutrition, hydration, etc.) can protect athletes and amateurs alike from infection by supporting post-exercise regeneration. This is especially important in the autumn when an increased number of illnesses in the general population and unfavorable environmental conditions are noted. It is also important to consider other stress factors that affect immunology, such as physical overload of the body or stress in private life.

### 4.2. Predictors of VO2 Max

The regression results for VO2 max in the test model show that the two principal components (CRP and hemoglobin) explained 94% (*R*^2^ adj.) of VO2 max variance. The significance probability level (*p* < 0.05) confirmed the statistical significance of the model, and the mean difference between the actual values of the dependent variable and the values predicted by the model was 8.63 (SE). Based on this, if CRP increases by one unit (assuming no change in the hemoglobin), the VO2 max decreases by 0.41 mL/kg/min. A one-unit increase in hemoglobin with no change in CRP will increase VO2 max by 2.35 mL/kg/min.

The results of the regression analysis for V02 max (all data) show that the resulting model including the two main components (ESR and platelets) explained 90% (*R*^2^ adj.) of the variance in the variable VO2 max. The mean difference between the actual values of the dependent variable and those predicted by the model was 7.61 (SE). The included predictors reached significance, but the overall model did not (*p* = 0.25). Based on the model presented, a limited estimate can be made that if ESR increases by one unit, with platelets unchanged, VO2 max will decrease by 0.51 mL/kg/min.

A one-unit increase in count platelets with no change in CRP will decrease VO2 max by 0.03 mL/kg/min.

The first model may also explain the prediction of VO2 max better than the second model because of R = 0.94, but it is worth discussing both.

In both models, the predictors were related to hemoglobin variables and factors indicative of the inflammatory process. ESR, platelets, and CRP decrease relative VO2 max (if the values increased, relative VO2 max decreased). Hemoglobin had the opposite effect. In response to an increase in its concentration, an increase in VO2 max is predicted.

Mathematical models attempt to characterize and evaluate the effects of a single exercise or training session. Different types are used depending on the availability and quality of data. Modeling was used to identify predictors of cardiovascular capacity in amateur cross-country skiers during the transition period. This study showed that in the earlier part of the annual training cycle, after de-training, the determining factors for VO2 max were monocytes, sodium concentration, and total calcium [39]. These studies have identified other predictors. Therefore, it seems important to monitor the health of amateurs at different times due to the variation and type of exercise performed at each stage. Such an assessment may be more accurate and better able to support an increase in exercise capacity.

The physiological and somatic determinants of VO2 max were also assessed using principal component analysis [40]. The mixed model used in McLaughlin et al.’s study [41] showed three variables affecting athletes’ performance (VO2 max, percentage of VO2 max on lactate threshold (%VO2 max LT), and running economy). This research did not asses these variables, so this is knowledge that should be included in future research.

Modeling and the use of statistics can be helpful in the case of sports training. However, it is worth considering the limitations of this approach. This is the impossibility of fully assessing all the factors that can determine a prediction. Random situations, for example, are still unpredictable. Nevertheless, the results of this research fill a gap in the knowledge of the determinants of VO2 max in a group of amateurs during the preparation period, after the basic preparation sub-period.

If athletes do not pursue adequate nutrition, sleep, and recovery, and do not include high-intensity units in their workload after the preparation period and at the start of the control period (with an increased incidence of illness in the population), inflammation and immunosuppression may be exacerbated. This would be equivalent to a decrease in VO2 max, as indicated by the regression in this study. It is therefore particularly important to adjust exercise loads appropriately on an individual basis and to monitor the biochemical response (indicators of inflammation and its persistence). Indirect methods of assessing exertion, such as the RPE (rating perceived exertion) scale, well-being assessment, orthostatic test, and heart rate monitoring, can also be helpful.

### 4.3. Limitations of the Study and Prospects

The monitoring of biochemical changes in response to exercise and training can be used to predict variations in the exercise capacity of amateur athletes. The results of these studies indicate that modifications in VO2 max can be anticipated in conjunction with variations in inflammatory factors and hemoglobin concentration. In instances where lower limit values are monitored, and these are approaching the lower reference ranges for the population, adjustments to the training plan and dietary regimen may prove beneficial. A reduction in the exercise load (in training intensity and volume) to the levels recommended for health in the population (primarily moderate intensity that does not disturb homeostasis or according to the recommendations of the World Health Organization) or the introduction of a pause in training may prove beneficial in maintaining VO2 max and protecting against immune depression and its consequences. In the case of hemoglobin, an increase may result from alterations in nutritional intake and supplementation, as well as the limitation of inflammatory changes, particularly in the intestines (through dehydration or intense exercise and restriction of blood flow through the intestines). Other variables identified as correlated with VO2 max in this study also highlight the necessity for monitoring biochemical variables in the cohort of amateur cross-country skiers.

The limitations of the study were related to the small number of participants. In the future, it would be valuable to increase the size of the group. It would be helpful to include other athletes in the research group, including women, with similar exercise capacity, and professional athletes or inactive people (control group). The assessment of markers of myocardial damage and echocardiography would also be helpful. This would also be valuable in statistical evaluations and the determination of regression models.

Further research could also enhance our understanding of the influence of physical exercise and training on immunological variables and hemoglobin in amateur athletes. Furthermore, an assessment of the types of immunoglobulins, including those present in saliva, could be conducted. Furthermore, it would be beneficial to conduct studies at three-month intervals at different stages of the annual training cycle. Furthermore, an assessment of diet and nutritional behavior would undoubtedly prove beneficial. The findings of such studies could serve to corroborate the existing results and their implications. Nevertheless, it would be prudent to consider enlarging the sample size in future studies, with a particular focus on including women and individuals with specific health conditions, such as obesity or metabolic disorders. This approach would undoubtedly enhance our comprehension of the influence of cross-country skiing on the health of amateur marathon competitors.

## 5. Conclusions

The study aimed to determine the biochemical factors influencing cardiovascular performance in male long-distance ski amateurs during the preparation period. Nine biochemical variables were correlated with VO2 max. Two regression models showed two determinants each, these being hemoglobin (positive influence) and CRP (negative), as well as ESR and platelets (negative). The results show that amateur athletes should have their inflammation and erythrocyte system assessed. There is still a lack of knowledge about changes in various parameters in middle-aged amateur athletes, and this research provides valuable insights to fill that gap. Biochemical monitoring and assessment are essential tools for evaluating the individual adaptation to exercise and developing an effective training plan. The application of this knowledge, along with the subsequent changes, can facilitate the attainment of optimal individual performance capabilities among cross-country skiing amateurs.

## Figures and Tables

**Table 1:** The Pearson correlation coefficients were used in the case of statistical significance.

Coefficient (*r*)	Correlation
0.0 ≤ |*r*| ≤ 0.2	no
0.2 < |*r*| ≤ 0.4	weak
0.4 < |*r*| ≤ 0.7	average
0.7 < |*r*| ≤ 0.9	strong
0.9 < |*r*| ≤ 1.0	very strong

**Table 2:** VO2 max and somatic variables of the participants.

Variable	Mean ± SD
Age (years)	37.87 ± 6.58
Height (cm)	181.44 ± 6.53
Body mass (kg)	78.38 ± 6.01
Fat mass (kg)	11.44 ± 2.75
Fat mass (%)	14.57 ± 3.04
BMI (kg/m^2^)	23.58 ± 1.17
VO2 max (mL/kg/min)	51.08 ± 4.61

BMI: body mass index. VO2 max: maximal oxygen uptake. SD: standard deviation.

**Table 3:** The *p*-values and correlations for the VO2 max of the athletes.

Variable	*p*-Value	Correlation
Leukocytes (thou/µL)	0.036	0.370
Erythrocytes (M/µL)	0.042	0.184
Hemoglobin (g/dL)	0.005	0.447
Hematocrit %	0.016	0.469
Platelets (thou/µL)	0.043	−0.397
Procalcitonin (PCT) %	0.043	−0.272
Erythrocyte sedimentation rate (ESR) (mm/h)	0.017	−0.507
Gamma-glutamyl transferase (GGTP) (U/L)	0.009	−0.415
C-reactive protein (CRP) (mg/dL)	0.004	−0.391

**Table 4:** The best model for V02 max—test data. *R*^2^ = 0.94.

Variable	Regression Coefficient	Statistical Error	*t*-Value	*p*-Value
Intercept	−20.59	8.63	−2.39	0.04
CRP (mg/dL)	−3.35	0.92	−3.65	<0.001
Hemoglobin (g/dL)	2.35	0.40	5.90	<0.001

**Table 5:** The best model for V02 max—training set data. *R*^2^ = 0.90.

Variable	Regression Coefficient	Statistical Error	*t*-Value	*p*-Value
Intercept	9.16	7.61	1.20	0.25
Erythrocyte sedimentation rate (ESR) (mm/h)	−0.51	0.12	−4.11	<0.001
Platelets (thou/µL)	−0.03	0.01	−2.58	0.03

## Data Availability

Data are available on request due to privacy and ethical restrictions.
